# CGRP: A New Endogenous Cell Stemness Maintenance Molecule

**DOI:** 10.1155/2022/4107433

**Published:** 2022-01-29

**Authors:** Xiaoting Lv, Qingquan Chen, Shuyu Zhang, Feng Gao, Qicai Liu

**Affiliations:** ^1^Department of Respiratory and Critical Care Medicine, Research Laboratory of the Respiratory System Diseases, 1st Affiliated Hospital, Fujian Medical University, Fuzhou, Fujian 350005, China; ^2^Department of Laboratory Medicine, School of Medical Technology and Engineering, Fujian Medical University, Fuzhou, Fujian 350004, China; ^3^Clinical Laboratory, Fujian Maternity and Child Health Hospital, Affiliated Hospital of Fujian Medical University, Fuzhou, Fujian 350005, China; ^4^Department of Pathology, 1st Affiliated Hospital, Fujian Medical University, Fuzhou, Fujian, 350005, China; ^5^Department of Reproductive Medicine Centre, 1st Affiliated Hospital, Fujian Medical University, Fuzhou, Fujian 350005, China

## Abstract

Stem cells have the ability of self-replication and multidirectional differentiation, but the mechanism of how stem cells “maintain” this ability and how to “decide” to give up this state and differentiate into cells with specific functions is still unknown. The Nobel Prize in physiology and medicine in 2021 was awarded to “temperature and tactile receptor,” which made the pain receptor TRPV1-calcitonin gene-related peptide (CGRP) pathway active again. The activation and blocking technology of CGRP has been applied to many clinical diseases. CGRP gene has complex structure and transcription process, with multiple methylation and other modification sites. It has been considered as a research hotspot and difficulty since its discovery. Drug manipulation of TRPV1 and inhibition of CGRP might improve metabolism and prolong longevity. However, whether the TRPV1-neuropeptide-CGRP pathway is directly or indirectly involved in stem cell self-replication and multidirectional differentiation is unclear. Recent studies have found that CGRP is closely related to the migration and differentiation of tumor stem cells, which may be realized by turning off or turning on the CGRP gene expression in stem cells and activating a variety of ways to regulate stem cell niches. In this study, we reviewed the advances in researches concentrated on the biological effects of CGRP as a new endogenous switching of cell stemness.

## 1. Introduction

Stem cells are a kind of cells with the ability of self-replication and multidirectional differentiation, which can differentiate into any cells in the body. However, how stem cells preserve this ability and “decide” to give up this state and transform into specific cells are two questions perplexing scientists. Successfully answering these two questions will greatly expand our ability to use stem cells to treat diseases. There are many specific open DNA regions in stem cells, which help stem cells produce many proteins to prevent stem cells from self-differentiation. Once stem cells begin to differentiate into a specific type of cells, these specific open DNA region fragments will be closed due to a variety of regulatory mechanisms, such as methylation, transcription factors, enhancers, and silencers [1–3], the pattern that stem cells begin to differentiate only by reading specific DNA regions. The regulation mechanism of the “selective silencing” process needs to be deeply studied, and the molecular switch of cell stemness is the mainstream direction in the current research. In the past, transcription factors of OCT4, SOX2, and NANOG were considered to be the stemness-related molecular switches for embryonic stem cells. That is, as the expression of OCT4, SOX2, and NANOG was increased, self-renewal genes were turned on and differentiation genes were turned off. When OCT4, SOX2, and NANOG express, self-replication genes were turned on while differentiation genes were turned off. These three transcription factors could interact to regulate the downstream target genes for maintenance and self-differentiation of embryonic stem cells, which allowed stem cells to renew themselves without input signals [4–6]. In addition, OCT4, SOX2, and NANOG transcription factors have been hotspots in recent research of tumor stem cells [7, 8], yet their research in other stem cell differentiation fields is extremely limited, which is still far from clinical application. Therefore, it is necessary to explore other potential cell stemness switching molecules.

Calcitonin gene-related peptide (CGRP) is a bioactive peptide composed of 37 amino acids, which is mainly distributed in C and A*δ* sensory fibers of the peripheral and central nervous system (CNS) and closely connected with peripheral blood vessels [9–12]. As an important member of the neuropeptide family, CGRP is expressed in a variety of body fluids, such as plasma [13, 14], saliva [15], tears [16], knee synovial fluid [17], and cerebrospinal fluid [18, 19]. CGRP in the human body mainly includes two isomers: *α*-CGRP and *β*-CGRP, which are encoded by CALCA and CALCB, respectively [20]. Among them, *α*-CGRP is mainly distributed in the central and peripheral nervous systems [21, 22], while *β*-CGRP is mainly distributed in the brain, intestine, and thyroid [23]. The consistency between *α*-CGRP and *β*-CGRP sequences is more than 90%, and there is only one amino acid difference between these two isomers [24].

**Figure 1:**
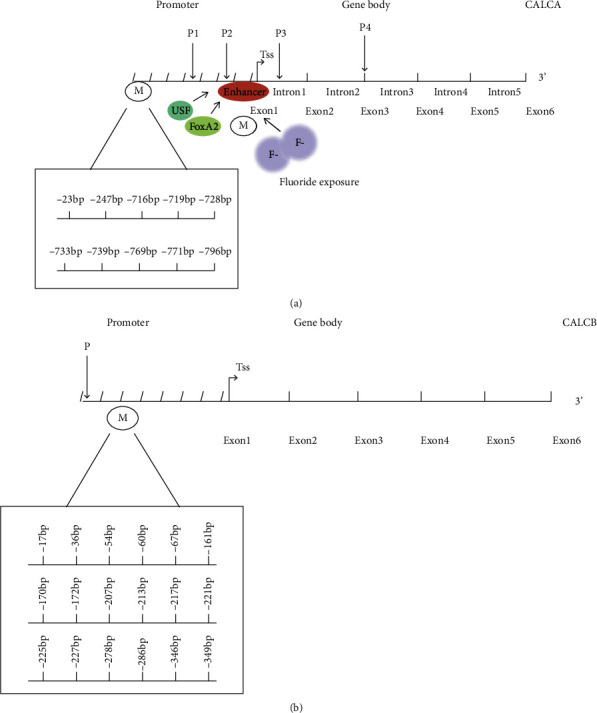
Diagram of the structures for both the CALCA and CALCB genes. Both the CALCA and CALCB genes contain all six exons, with multiple methylation and other modification sites. (a) Multiple methylation sites of CALCA promoter with a large area of CpG in the 5′ end were found in different types of bacterial preterm sepsis (-716 bp, -719 bp, -728 bp, -733 bp, -739 bp, -769 bp, -771 bp, and -796 bp) and pancreatic ductal adenocarcinoma and its paracancer (-23 bp, -247 bp), and the methylation of CALCA exon 1 was related to fluoride exposure. CALCA enhancer could also be activated by upstream stimulating factor (USF) and forkhead protein Foxa2. Four novel polymorphic alleles were found in neurological or psychiatric disease: two (g.979G>A and g.4218T>C) represented single nucleotide polymorphisms (SNPs), one consisted of two coupled SNPs in close vicinity to each other (g.1210T>C and g.1214C>G), and one was an intronic 16 bp microdeletion (2919-2934del16). One of the SNPs (g.4218T>C) causes a nonsynonymous amino acid change (Leu66Pro) in the third exon, an exon common to both procalcitonin and pro-a-CGRP. (b) Multiple methylation sites of CALCB promoter with a large area of CpG in the 5′ end were found in pancreatic ductal adenocarcinoma and its paracancer (-17 bp, -36 bp, -54 bp, -60 bp, -67 bp, -161 bp, -170 bp, -172 bp, -213 bp, -217 bp, -221 bp, -225 bp, -227 bp, -247 bp, -278 bp, -286 bp, -346 bp, and -349 bp). Promoter polymorphisms of CALCB gene (rs11603873 T/C and rs79501047 A/G) are common in the Han population, and the rs11603873 C genotype has a high risk of salivary adenoid cystic carcinoma compared with the rs11603873 T genotype.

The posttranscriptional process and shearing process of CGRP gene are diverse and complex, which is strictly controlled in a specific way. CGRP gene transcription was activated or inhibited by different tissue-specific factors. CALCA transcripts are expressed differently in different tissues, such as calcitonin transcripts in thyroid C cells and *α*-CGRP transcripts in nerve cells, and CALCB transcripts were also expressed only in specific cell lines [23]. CALCA is transcribed into a primary transcript mRNA precursor containing six exons and then cut into three different secondary transcripts (Figure 1(a)): (1) *α*-CGRP: mature CGRP mRNA is formed by cutting special introns containing exons 1, 2, 3, 5, and 6. Among them, exons 1 and 6 are noncoding RNA, and exons 2, 3, and 5 are the gene basis of *α*-CGRP polypeptide precursor in translation. The macromolecular polypeptide precursor is hydrolyzed by protease: signal peptide related to exon 2 is further cleaved. The N-terminal polypeptide related to exon 3 and CGRP related to exon 5 mainly are produced in nerve cells. (2) Calcitonin (CT): it is cut into mature CT mRNA composed of exons 1, 2, 3, and 4. During translation mainly occurring in thyroid cells, CT precursor protein is hydrolyzed by protease into the following peptides: signal peptide, N-terminal polypeptide, and C-terminal polypeptide, which is called carboxyl terminal polypeptide 1 (ccp-1). The *α*-CGRP and CT transcripts had three common exons, but contained different 3′ ends. (3) Recently, a third mode of CT/CGRP mRNA formation has been found. This mature CT mRNA II contains 6 exons. During translation mainly occurring in B cells, the CT precursor protein with different amino acid sequence from CT precursor protein is formed, which is hydrolyzed by protease to release CCP-1. In addition, the increased concentration of N-terminal and C-terminal regulatory peptides could negatively inhibit the expression of CT/CGRP gene. b-CGRP encodes only one transcript, but contains a potential 3′ splice site and a fragment similar to calcitonin exon, called “degenerate exon.” Although the splicing of this “residual” exon has never been observed, the Bgl I1 site in this fragment can be inserted into different fragments of CGRP gene to construct CALCA gene and CALCB gene chimera (Figure 1(b)) [23].

In addition to the known biological functions of relaxing blood vessels and relieving pain, CGRP is also involved in the regulation of immune response and cell proliferation and apoptosis after tissue injury [9, 12, 25, 26]. What is more, CGRP has been proven to be an important neuroendocrine regulator of human longevity. It is known that the antagonistic effect of transient receptor potential cation channel subfamily V member 1 (TRPV1)+ neurons on the insulin secretion of pancreatic B cells and the negative impact on metabolism may be achieved by stimulating the sustained high level of CGRP, and the CGRP homeostasis is destroyed with aging, resulting in the decline of metabolism [27]. More importantly, CALCA and CALCB, which were highly expressed in undifferentiated pluripotent stem cells, have been proven to play a regulatory role in stem cell differentiation as immune privilege genes [28]. During the physiological process of tissue injury repair, it was found that CGRP+ sensory nerve fibers were reinnervated. High expression of CGRP could not only promote the migration of mesenchymal stem cells (MSCs) to damaged tissues [29] but also promote stem cell differentiation [30, 31]. CGRP is also considered as a tumor growth factor to induce the occurrence and development of malignant tumors [32, 33]. Therefore, the abnormal expression of CGRP gene can regulate the ability of differentiation and self-renewal in stem cells.

The expression of CGRP gene can be regulated at the transcriptional level. DNA methylation is the most widely studied content of epigenetics. It mainly studies the regulation of gene expression at the transcriptional level without DNA sequence change, and this change can exist and inherit stably during DNA replication. Cytosine residue methylation is the most widely studied epigenetic modification, which mostly exists in CG sequence [34]. Different elements such as promoter, gene ontology, enhancer, silencer, and transposon in genome can be methylated and affect gene expression [35]. About half of the genes in vertebrates are enriched in CpG sequences on the promoter, which is called CpG island. The DNA methylation level of the promoter with CpG island can be closely related to the transcriptional activity of the gene by binding to transcription factors or changing chromatin structure [36]. It also reflects species specificity [37]. CALCA has a 5′ flanking sequence of more than 1.8 kb. A large area of CpG island has been found in the 5′ end promoter (Figure 1(a)). It is rich in CpG, has high content of G+C, and contains several rare restriction endonuclease digestion sites and two different CpG-rich regions, one around exon 1 and another in about 1.5 kb upstream in a nonmethylated state generally [38]. DNA methylation regulates cell proliferation, apoptosis, and gene expression, which is closely related to tumor [33, 39]. Abnormal methylation levels of CALCA promoter and exon can change cell stemness [35, 40]. Methylation of CALCA promoter in bone marrow cells is often observed in patients with myelodysplastic syndrome (MDS) so demethylated drugs can delay the progression of MDS [41]. In addition, DNA methylation is one of the regulatory mechanisms on gene tissue-specific expression [42]. The degree of DNA methylation is regulated by microenvironment, which is also called stem cell niches [43, 44]. Both CALCA and CALCB have multiple methylation sites (Figure 1). So the promoter region of CALCA gene in different subtypes of the same disease could have methylation changes at different sites [45]. Thus, the methylation level of CGRP gene could be used as a biomarker for diagnosis and treatment in many clinical diseases. The use of demethylated drugs in the microenvironment of stem cells might also benefit patients.

At present, the function of CGRP in stemness-related fields is not clear. The goal of this review is to provide an overview about regulation mechanism of CGRP pathophysiology, with an emphasis on the biological effects on cell stemness in different tissues.

## 2. CGRP and Lung Stem Cells

### 2.1. CGRP and Lung Stem Cells and Their Microenvironment

Lung epithelial stem cells are considered to be facultative stem cells. Namely, it is in a resting state and participates in the process of lung breathing, secreting proteins that are crucial to gas exchange normally. However, they will become transient expansion cells and differentiate into one or more highly differentiated cells quickly in regeneration and repair after injury [46]. Lung epithelial stem cells include basal cells (BCS) (basal stem cells (BSCs) and basal lumen progenitor cells (BLPCs)) of proximal airway (trachea bronchus), ductal cells, Clara cells of bronchioles and pulmonary neuroendocrine cells (PNECs), distal airway stem cells (DASCs), and alveolar epithelial stem cells such as type II alveolar cells (AT II). In the early embryo (embryonic stage 13-15 days (E13-E15)), CGRP is positive by immunostaining in all epithelial cells of the distal airway, but it is limited to different cell lines such as PNECs at the late embryonic stage [47]. The transient expression of CGRP in different differentiated cell types in stem cells indicates that CGRP may modulate the activity of stem cells.

Stem cell niches, composed of different types of cells, extracellular matrix proteins, and growth factors, are specialized microenvironments that promote the maintenance of stem cells and regulate their function [48]. Stem cell niches play a key role in determining the function and differentiation efficiency of stem cells [44, 49]. For example, tracheal submucosal secretory glands (SMGs) provide a survival microenvironment for airway stem cells, and the mucus secreted by niches is not only related to congenital immunomodulation but also contains the substances which can maintain the pluripotency of epithelial stem cells and provide local environmental signals to maintain and mobilize stem cells [50]. Thus, the occurrence of a variety of hypersecretive lung diseases such as chronic bronchitis, asthma, and cystic fibrosis may be related to the abnormal proliferation and differentiation of stem cells in SMGs [50].

**Figure 2:**
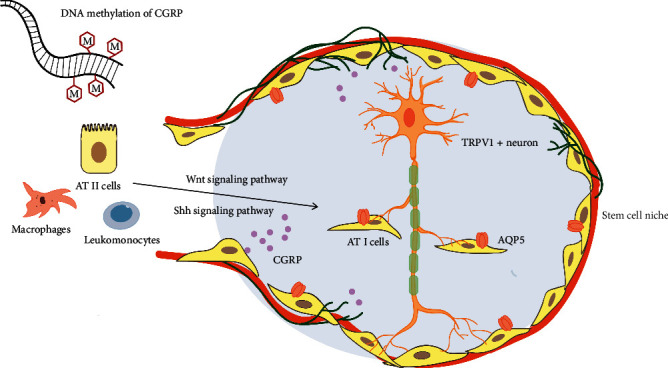
CGRP secreted by TRPV1 neurons in the stem cell niches promotes the proliferation and differentiation of AT II cells. Stem cell niches are specialized microenvironment composed of different types of cells, extracellular matrix proteins, and growth factors. Promoter DNA methylation regulates CGRP expression. CGRP secreted by TRPV1 neurons in the stem cell niches takes effect on lung pluripotent stem cells to induce the proliferation and differentiation of AT II cells through downstream pathways, such as Sonic hedgehog signaling pathway and Wnt signaling pathway.

The function of SMGs was regulated by sympathetic or parasympathetic nerves. There are abundant parasympathetic neurons with transient receptor potential vanilloid 1 (TRPV1) membrane protein in the tracheal mucosal layer, and the CGRP released by TRPV1+ neurons is the main source of CGRP [51, 52]. In stable conditions, CGRP expression maintained a low level and is increased after lung injury. Only high secretion of CGRP was detected in patients with hereditary cystic fibrosis in the submucosal secretory gland of the trachea, while the levels of other neurotransmitters such as VIP and SP did not change significantly, which confirmed that CGRP was the main neurotransmitter [52]. Although the elevated CGRP level can stimulate the proliferation of mouse SMG progenitor cells [52], it is speculated that CGRP has limited effect on slow circulating stem cells due to the short expression of CGRP [51]. Therefore, CGRP secreted by TRPV1 neurons in the stem cell niches may take effect on lung pluripotent stem cells through downstream pathways to induce the proliferation and differentiation of stem cells [53–56] (Figure 2).

### 2.2. The Downstream Pathway of Lung Stem Cells Regulated by CGRP

#### 2.2.1. Sonic Hedgehog Signaling Pathway

Sonic hedgehog (SHH) signaling pathway is a major regulator of cell proliferation, cell differentiation, and tissue polarity [57]. In multiple adult tissues, it becomes active during differentiation, proliferation, and maintenance by regulating cell stemness in the process of embryonic development [58, 59]. Abnormal expression of the SHH pathway may cause severe lung dysplasia [60, 61].

Pulmonary fibrosis is characterized by epithelial mesenchymal transition (EMT) in epithelial lesions. Highly differentiated AT I cells with limited repair ability cover more than 95% of the alveolar surface, meaning that it is more vulnerable to be damaged [62–64]. Adamson et al. found that alveolar stem cells (AT II cells) could migrate and proliferate to the alveolar surface after mild alveolar injury [62, 65, 66]. At the same time, it promoted the transformation of activated fibroblasts into quiescent state and inhibited TGF-*β* thus limiting lung fibrosis [67]. However, if the alveolar epithelium was damaged more seriously or the ability of proliferation of AT II cells was impaired, it will lead to lung fibrosis [68, 69]. It was found that the expression of SHH, smoothened (Smo), patched (Ptch), and Gli1 pathway proteins was increased significantly in idiopathic pulmonary fibrosis (IPF), suggesting that the SHH pathway may accelerate pulmonary fibrosis via alteration of self-renewal and differentiation of lung stem cells [70–72]. It was shown that CGRP could protect AT II cells from hyperoxia-induced damage through the SHH signaling pathway [73] (Figure 2). Further study will focus on the relationship of CGRP and SHH pathway on the proliferation and differentiation of lung stem cells in pulmonary fibrosis.

#### 2.2.2. Wnt Signaling Pathway

The Wnt signaling pathway is also considered to be one of the key cascades that regulate development and stem cells and closely related to maintenance, metastasis, and immunity in the tumor stem cells [74]. After alveolar injury, AT II cells clonally proliferated as alveolar stem cells and then transdifferentiated into AT I cells [75]. It was found that the Wnt7b/*β*-catenin signaling pathway was involved in the protective action of CGRP on hyperoxia-induced lung injury and CGRP inhibitor CGRP8-37-induced obvious inflammation with damaged AT II cells and alveolar structural remodeling in lung tissue [76].

The transdifferentiation of AT II cells may be regulated by CGRP and Wnt signaling pathway in pulmonary fibrosis. Recently, it has been found that the imbalance of water and sodium in alveolar epithelial cells could promote apoptosis and enhance epithelial mesenchymal transformation. That is to change ATII cell stemness and promote pulmonary fibrosis [77]. Aquaporin 5 (AQP5), located in the membrane of alveolar epithelial cells, is a member of the AQP family which participates in maintaining water electrolyte balance. Usually, AQP5 is considered as the differentiation marker of AT I cells [77, 78]. In acute lung injury, it was confirmed that the level of AQP5 in the early stage of inflammation was negatively correlated with the apoptosis of AT I [78, 79]. Our research has found that alveolar epithelial cells were widely apoptotic and the transdifferentiation ability of alveolar stem cells AT II was impaired in the process of pulmonary fiber. At the same time, the imbalance of *β*-CGRP/*α*-CGRP regulated AQP5 expression and promoted transition of alveolar epithelial cell apoptosis via the TGF-*β*/P-smad1/Smad4 pathway leading to a decreased AQP5 expression in pulmonary fibrosis [80]. In vitro experiments also confirmed that Wnt7a was a ligand of AQP5, the differentiation marker of AT I cells, and the Wnt pathway can affect the transdifferentiation of AT II cells to AT I cells by regulating the expression of AQP5 [75]. Therefore, treatment with AT II cells can accelerate the repair of alveolar epithelium, which may be expected to be used to repair lung injury such as lung fibrosis [81].

### 2.3. Promoter DNA Methylation Regulates CGRP Expression

CGRP can possess multiple methylation sites via complex processing procedures. In different types of bacterial preterm septicemia, CpG sites in the promoter region of CALCA gene can have four different DNA methylation patterns, such as -769 bp, -771 bp, and -778 bp CpG mutation (Figure 1(a)). Different DNA methylation sites in different infection types lead to different expression levels of CGRP, suggesting its potential role as epigenetic biomarkers and demethylation therapy [45].

## 3. CGRP and Bone Stem Cells

### 3.1. CGRP and Hematopoietic Stem Cells

Stem cell regulation and classification of human bone stem cells remain largely unexplored [82]. The most well-characterized stem cells in the skeleton is the hematopoietic stem cell (HSC), defined by its functional ability to form colonies in vitro and serially reconstitute long-term hematopoiesis in conditioned hosts [83].

CGRP treatment in mice can reduce the number of IL-7-reactive B cell progenitor cells in the bone marrow. The reduction is dose-dependent and can be blocked by CGRP receptor antagonist CGRP8-37. Even a single injection of CGRP still has a long-term effect on B cell development [84]. The stem cells niche in which HSCs are located could also be affected by CGRP. Studies have shown that CGRP+ nociceptive nerves were necessary for HSC mobilization, induced by CGRP driving granulocyte colony-stimulating factor (G-CSF), suggesting that CGRP+ nociceptive nerves were interacted with sympathetic nerves to regulate HSC niches and maintain the function of HSC in the bone marrow [85–89]. Unlike the sympathetic nerves that regulate HSC through niches indirectly [85, 86, 88], parasympathetic nerves act on HSC through RAMP1 and calcitonin receptor-like receptor (CALCRL) directly and promote CGRP excretion by activating the Gas/adenylate cyclase/cAMP pathway (Gas/AC/cAMP pathway). And then capsaicin, a natural component of pepper, can trigger the activation nociceptive neurons to enhance the HSC mobilization in mice via releasing CGRP [89].

### 3.2. CGRP and Mesenchymal Stem Cells

Mesenchymal stem cells (MSCs), the nonhematopoietic lineages in skeletal tissue, are a kind of mesodermal pluripotent stem cells, which mainly exist in the bone marrow [82]. Recent studies have shown that the CGRP gene can be stably expressed in MSC after transfection with recombinant CGRP lentivirus. And CGRP recombinant lentivirus transfection has little effect on the proliferation and aging of bone marrow mesenchymal stem cells [90]. The bone MSCs of rats modified by CGRP might inhibit the proliferation and migration of rat vascular smooth muscle cells (VSMCs) [90]. These results lay a foundation for the application of CGRP-modified bone marrow mesenchymal stem cells in the treatment of vascular restenosis.

CGRP could induce osteogenic differentiation of bone marrow MSCs after skull defect [91]. The number of MSCs in knee synovial fluid was increased after traumatic meniscus injury, while MSCs were scarcely discovered in degenerative meniscus injury. So CGRP in synovial fluid after trauma were speculated that it could also induce MSCs to enter synovial fluid from the synovium [29]. The sensory neurons (SNs) that could release CGRP in the early to middle stage of bone integration could remodel endothelial cell extracellular matrix, enhance angiogenesis, and induce bone mesenchymal stem cells to differentiate into osteoblasts [92, 93]. It was suggested that before periosteal vascularization, ossification, and mineralization of ulna fracture, the prolification and dendrization of CGRP+ TrkA+ sensory nerve fibers in reactive periosteum were obvious in the cell domain with a variety of nerve growth factor (NGF), meaning that reinnervation of CGRP+ sensory nerve fibers could promote bone stem cell differentiation [30]. The CGRP level in the blood of patients with fracture was increased significantly, and the CGRP-positive nerve fibers were also been found clearly at the fracture sites in the early stages of fracture [94, 95]. *α*-CGRP is closely related to the osteogenic differentiation of stem cells. Bone remodeling in CALCA knockout mice appeared to be a significant problem, while mice lacking *β*-CGRP have been shown to display only a mild and temporary problem [22, 95, 96].

The mechanism of CGRP promoting osteogenic differentiation in stem cells could be demonstrated by the following four aspects. Firstly, *α*-CGRP enhanced osteogenic differentiation of stem cells by inducing calmodulin- and RAMP1-dependent activation of cAMP response element-binding protein 1 (CREB1) and Sp7 (also known as osterix) [97]. RAMP1 could also promote CGRP-induced osteogenic differentiation of BMSCs by regulating the Hippo/Yap pathway [98–101]. Moreover, magnesium ion can help CGRP mediate osteogenic differentiation of stem cells in newborn bone [97]. Magnesium ion can induce the formation of new bone, and magnesium deficiency will lead to osteoporosis. Magnesium ion can stimulate the release of *α*-CGRP, promoting the osteogenic differentiation of stem cells in the periosteum. If sensory nerves were destroyed or periosteum with a large number of sensory nerves was removed, magnesium-induced bone formation is greatly reduced [97, 102]. Thirdly, it was demonstrated that CGRP administration not only stimulated osteoblast differentiation but also inhibited OPG/RANKL-regulated osteoclastogenesis [103]. Finally, after distraction osteogenesis (DO), CGRP significantly increased the proportion of endothelial progenitor cells (EPCs) and capillary density. In addition, the endothelial differentiation of MSCs was induced and the number of endothelial progenitor cells (EPCs) by activating the PI3K/Akt signaling pathway was increased in defected bone area [104]. CGRP may act as a modulator of bone metabolism through osteoblast- and osteoclast-associated mechanisms, which result in osteoblast formation with subsequent activation of bone formation.

### 3.3. Abnormal Methylation Levels of CALCA Promoter and Exon Change Function of Stem Cells

It is known that excessive exposure to fluoride affected bone mineral density (BMD). Fluoride exposure can upregulate the methylation level of CALCA exon 1 and inhibit the expression of CT in Chinese women, resulting in the decrease of bone mineral density [40]. After alveolar bone destruction caused by periodontitis, CGRP can promote bone tissue repair, growth, and metabolism to realize bone tissue regeneration (Figure 1(a)) [105], which may be achieved by affecting the function of dental pulp stem cells (DPSCs). As Chinese toothpaste products generally contain fluoride, we speculate that fluoride may reduce bone mineral density by the CALCA methylation, causing alveolar bone destruction and blocking the regeneration process of periodontal alveolar bone.

Studies showed that adipose-derived stem cells (ASCs) in patients with type 2 diabetes mellitus (T2DM) caused upregulation of methylation level in promoter region of CALCA target fragment, resulting in a significant reduction in the osteogenic function of stem cells [35]. The above studies indicated that the abnormal methylation level of CALCA segments in stem cells may change the cell stemness on bone stem cells.

## 4. CGRP and Skeletal Muscle Repair

CGRP signal can regulate the differentiation of muscle MSCs and muscle repair. Interleukin- (IL-) 33 produced by local MSCs in injured muscle stimulates Foxp3+CD4+ regulatory T (Treg) cells to promote the repair of acute or chronic injured skeletal muscle [106, 107]. IL-33+ muscle MSCs are surprisingly close to large fiber nerve bundles and small fiber sensory neurons, which can transcribe a series of genes of encoding neuropeptides, neuropeptide receptors, and other nerve-related proteins, including muscle MSC subtypes that simultaneously express IL-33 and CGRP receptors; CGRP signal can regulate the production of IL-33 by muscle MSCs and the subsequent accumulation of muscle dendrites [108]. In the early stage of acute skeletal muscle injury, a single injection of CGRP can induce bone marrow mesenchymal stem cells to produce a large number of genetic programs [108].

## 5. CGRP and Skin Stem Cells

Skin is a complex and highly regenerative tissue. There are many different types of stem cells, including epidermal stem cells, mesenchymal stem cells, endothelial and hematopoietic precursor cells, and neural crest-derived precursor cells. In vitro experiments confirmed that nearly 100% of induced pluripotent stem cells (iPSC) produced by human skin fibroblasts expressed neuronal markers, such as BRN3A and *β*3-tubulin. Its TRPV1 and neurofilament M were positive [109]. More importantly, CGRP could be released by IPSC-derived neurons when stimulated with molecules that induce neuropeptide release, suggesting that innervation plays a central role in many human skin diseases, such as psoriasis and atopic dermatitis [109]. Skin-derived precursor cells (SKPs) were induced to differentiate into sensory neurons (SNs) in vitro which expressed TRPV1, CGRP, PAR2, TRPA1, substance P, etc., meaning that CGRP is closely related to SKP differentiation [110]. In addition, the epidermal stem cells have been reported to be related to epidermal hyperproliferative diseases and skin tumors. Epidermal stem cells can not only maintain the stability of the homeostasis but also participate in tissue regeneration and repair. It is confirmed that CGRP promoted epidermal stem cells to enter S phase, stimulated epidermal stem cells to leave their niches, broke the static state, and induced differentiation [111].

On the other hand, the high level of plasma *α*-CGRP and *β*-CGRP and CGRP gene polymorphism has been found in patients of psoriasis and CALCA T-692C polymorphism TT genotype is a susceptible factor in patients with psoriasis vulgaris (Figure 1(a)) [112].

## 6. CGRP and Nervous System Diseases

### 6.1. CGRP and the Stem Cell Repair Process of Neurovascular Inflammation

CGRP triggered antiapoptotic signaling and released a number of neurotrophins (including insulin-like growth factor-1 (IGF-1), basic fibroblast growth factor (bFGF), and nerve growth factor (NGF)), increasing antioxidant defenses. It also upregulated the growth factor signals from endothelial cells to brain parenchyma, enhanced the blood-brain barrier, promoted stem cell niche neurogenesis and angiogenesis, and stimulated neurogenesis. Therefore, CGRP is involved in neuroprotective activation after extensive ischemia injury, hyperthermia, and seizure and activates neuroprotective processes [113]. Carriers of at least one C allele at the polymorphic site CALCA T-692C showed increased risk for hypertension [114]. The CALCB gene polymorphism rs16930880 was also confirmed to be associated with vasospasm of Reynolds phenomenon [115]. As the mice grew older, the aging lumbar dorsal root ganglia (DRGs) showed peripheral sensitization, inflammatory reaction, and high expression of neurotransmitter CALCA mRNA [116]. The high expression of CGRP is involved in the stem cell repair process of neurovascular inflammation. Therefore, it is speculated that the stem cell niches are related to the survival, proliferation, migration, and differentiation of stem cells, which may be beneficial for natural or therapeutic CGRP nerve regeneration.

### 6.2. CGRP Methylation and Migraine

CGRP is known as the strongest vasodilator. In the pathophysiology of migraine, it was related to neurogenic inflammation and cerebral vasodilation [117], neuropeptide CGRP, abundant in trigeminal ganglion neurons, released from peripheral and central nerve. It was interacted with adjacent neurons and satellite glial cells in trigeminal ganglion endocrine, making peripheral sensitization permanent, and it could drive central sensitization of secondary neurons, which may promote the progression of paroxysmal migraine to chronic migraine [118, 119].

There is a strong correlation between DNA methylation of migraine-related genes in migraine patients [120]. On the one hand, it was reported that CpG island methylation around the 18 bp enhancer was a key determinant for the specific expression of CALCA gene in trigeminal neurons [121, 122]. The enhancer of CALCA gene is located about 1 KB upstream in the transcription initiation site, and its methylation on the flank of the enhancer could induce trigeminal ganglion glial cells to activate and produce pro-CT and CGRP in vitro [123]. Upstream stimulating factor (USF) 1 and USF2 could combine with CGRP enhancer, inducing CALCA promoter activity in trigeminal neurons (Figure 1(a)) [122]. CALCA enhancer could also be activated by USF and forkhead protein Foxa2 [124]. On the other hand, transcriptional memory allows certain genes to respond to previously experienced signals more robust. Interestingly, it is proved that cytokine TNF-*α* transcriptional memory is related to methyl-labeled genes [125]. Also, coding as a therapeutic target for migraine, CALCB has high initial methylation level and CpG density around NF-*κ*B sites, which are correlated with the functional potential of transcriptional memory modules. TNF-*α*-mediated transcriptional memory is governed by active DNA demethylation of CALCB which makes memory genes extremely sensitive to many low-dose inflammatory signals [126].

The rs3781719 (T>C) single nucleotide polymorphism of 5-UTR in the promoter region of CALCA gene can also affect the expression of CGRP in the process of migraine [127]. The polymorphism of CALCA gene can affect the therapeutic effect of neuroblocker on a botulinum toxin A by changing the expression of CALCA gene [128]. Therefore, the treatment of interfering with the CGRP function of peripheral trigeminal nervous system is effective for migraine. Blocking the sensitization of trigeminal nerve by reducing the activity and level of peripheral CGRP may be effective to block the attack of migraine.

### 6.3. CGRP Gene Polymorphism and Other Neurological Diseases

Multiple polymorphisms of CALCA gene include two (g.979G>A and g.4218T>C) that represented single nucleotide polymorphisms (SNPs), one consisted of two coupled SNPs in close vicinity to each other (g.1210T>C and g.1214C>G), and one was an intronic 16 bp microdeletion (2919-2934del16). One of the SNPs (g.4218T>C) causes a nonsynonymous amino acid change (Leu66Pro) in the third exon, an exon common to both procalcitonin and pro-a-CGRP, which have been identified to be related to Parkinson's disease, schizophrenia, depression, and mania [129] (Figure 1(a)). The seemingly nonfunctional intron polymorphism could also disrupt normal RNA processing or introduce splicing sites into transcripts. It is reported that the 16 bp deletion in the first intron of CALCA gene matched the binding site of transcription factor AP-2 strongly expressed in neural crest-derived cells, and the deletion also eliminated the intron splicing enhancer (ISE) which might induce exon skipping [130].

## 7. CGRP and Tumor Stem Cells

When cancer cells undergo the EMT, they partially and transiently dedifferentiate. This transition provides an opportunity to adjust cellular gene expression and acquire a stem cell phenotype for self-perpetuation and propagation [131]. Some “negative” tissues and cell lines (such as malignant tumors) traditionally considered negative actually highly express CGRP, and some enzymes used for restricted CGRP expression could recognize CpG sites of some tumor cells [123, 132]. The possible mechanisms of obtaining cancer cell stemness by CGRP expression are as follows.

### 7.1. CGRP Regulates the Proliferation and Differentiation of Lung Tumor Stem Cells through the SHH Signaling Pathway

CGRP could regulate cell proliferation and apoptosis as a tumor growth factor by blocking the G0/G1 phase of the cell cycle to S phase and protect tumor cells from drug-induced apoptosis [133–135]. The SHH signal pathway plays a key role in the process of carcinogenesis and development, and its abnormal activation is closely related to the proliferation and differentiation of lung cancer stem cells (CSCs). Further studies show that the SHH signal pathway may be regulated by CGRP on the proliferation and differentiation of lung cancer cells [61, 136]. Pancreatic cancer cells could activate the SHH signaling pathway which increased the expression of CGRP and TRPV1 in the dorsal root ganglion in a concentration- and time-dependent manner, resulting in the pain of pancreatic cancer [137, 138]. In a word, whether CGRP regulates cell stemness through the SHH signaling pathway needs further study.

### 7.2. PNECs Regulate ILC2s through CGRP in Small Cell Lung Cancer

PNECs are rare cells with many neuronal characteristics in the body, having the secretory vesicles and the ability to sense environmental stimuli, and stem cells which can sense hypoxia and respond by differentiating into solitary NE cells secrete a protective peptide that mitigates hypoxic injury [139]. PNECs are considered as both progenitor cells and progenitor cell niches after airway epithelial injury [140]. In lung tissues, basal stem cells can directly differentiate into isolated PNECs triggered by hypoxia. Resection of these isolated PNECs could enhance epithelial damage, and administration of CGRP can save this excessive damage [139]. Thus, lung stem cells can reduce hypoxia damage by differentiating into PNECs which secrete CGRP under hypoxia condition.

Tumorigenesis is a complex process, which involves the interaction between cancerous cells and a variety of normal cells. Tumor is considered to be closely related to immunity. Small cell lung cancer (SCLC) is the most invasive type of lung cancer, characterized by poor prognosis and rapid resistance to treatments. The SCLC-like tumors growing subcutaneously in immunodeficient mice seemed to have low tumor potential (slow growth and noninvasive) [141]. PNECs are located near group 2 intrinsic lymphoid cells (ILC2s) near the airway branch point, and the function is still unclear [142]. CGRP is known to inhibit type 2 immune response in lung infection by inhibiting the production of type 2 cytokines in ILC2s in the lung [26]. CGRP released by PNECs can stimulate ILC2s and trigger downstream immune responses in bronchial asthma models [142]. Therefore, different microenvironment factors affect the neuroimmune function of PNECs, which may be related to tumor immune disorder.

**Figure 3:**
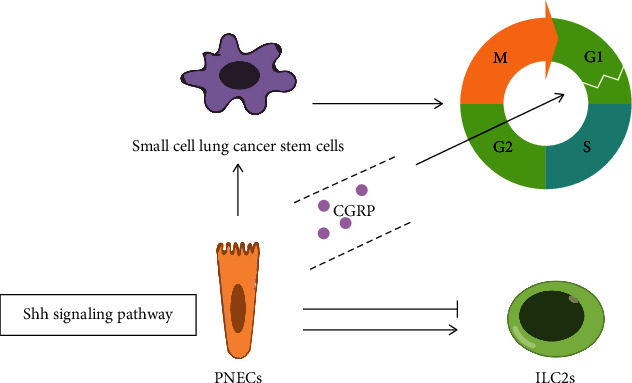
High CGRP expression promotes the malignant transformation of PNECs through the SHH signaling pathway. PNECs regulate group 2 innate lymphoid cells (ILC2s) through CGRP in small cell lung cancer which may affect the immune status of the tumor microenvironment. Moreover, CGRP protects stem cells from apoptosis by blocking the G0/G1 phase of the cell cycle to S phase through the SHH signaling pathway.

High expression of CGRP promotes the malignant transformation of small cell lung cancer target cell PNECs (Figure 3). Lineage tracing experiment shows that CGRP+ PNECs can not only self-renew in steady state but also differentiate into Clara cells and ciliary cells after lung injury [31]. SCLC is likely to originate from CGRP+ PNECs in neuroendocrine cells, consistent with their morphology and the neuroendocrine markers [143–146]. Inhibition of Notch signal can induce up to 10% of lung progenitor cells derived from human embryonic stem cells (hESCs) to produce PNEC and form early tumors similar to SCLC [141]. The expression of CGRP increased significantly during the carcinogenesis of PNECs [147, 148]. Therefore, it is of great significance to explore the mechanism of high CGRP expression promoting the malignant transformation of PNECs, and it also lays a foundation for the research of tumor therapy.

### 7.3. Trypsin Overexpression Induced Abnormal Splicing of CGRP in Pancreatic Cancer Stem Cells

The change of cell cycle is the driving force of tumor biological behavior [149], but whether it is unique in the role of cell cycle disorder in the occurrence of pancreatic cancer is still unknown. CGRP can induce G2/M phase arrest by regulating G2/M phase-related proteins [150]. Moreover, it is found that CGRP can arrest the cell cycle of pancreatic cancer stem cells in the G0/G1 stage [151]. Trypsin overexpression induced abnormal splicing of CGRP in pancreatic cancer stem cells, driving cell cycle disorder [152].

**Figure 4:**
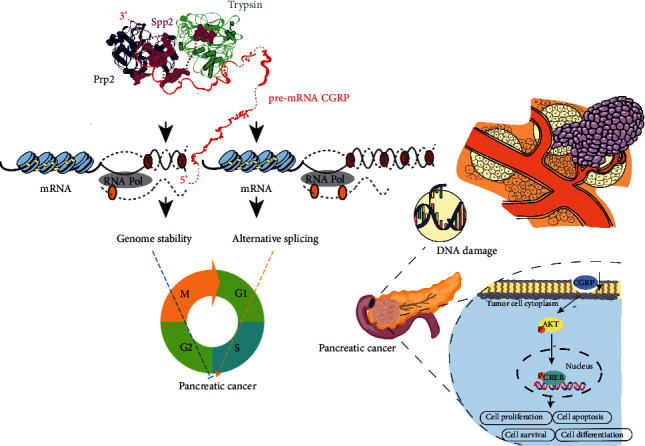
Abnormal CGRP expression promotes cell cycle disorder and promotes the occurrence of pancreatic cancer. The helicase Prp2 (blue) and the coactivator Spp2 (purple) combine stably with the anchor molecules on the spliceosome, so as to binding Prp2 to the activated spliceosome and allowing a greater role for Prp2. CGRP pre-mRNA (red) is loaded into the characteristic channel between the N- and C-halves of Prp2. The process of ATP binding and hydrolyzation or trypsin (green, specific high expression in the pancreas) triggers Prp2 to move into sensitive binding site of trypsin, driving pre-mRNA to move unidirectionally and gradually towards its 3′ end. By CGRP disturbing cell cycle, abnormal shearing breaks the genetic stability and amplifies oncogenic signals, whose function is similar to that of an essential translator and amplifier in the carcinogenic pathway.

However, CGRP does not complete the malignant cell transformation independently as Ras/Notch and other oncogenes in this process. The helicase Prp2 (blue) and the coactivator Spp2 (purple) combine stably with the anchor molecules on the spliceosome, so as to binding Prp2 to the activated spliceosome and allowing a greater role for Prp2. CGRP pre-mRNA (red) is loaded into the characteristic channel between the N- and C-halves of Prp2. The process of ATP binding and hydrolyzation or trypsin (green, specific high expression in the pancreas) triggers Prp2 to move into sensitive binding site of trypsin, driving pre-mRNA to move unidirectionally and gradually towards its 3′ end. By CGRP disturbing cell cycle, abnormal shearing breaks the genetic stability and amplifies oncogenic signals, whose function is similar to that of an essential translator and amplifier in the carcinogenic pathway [153–156]. Therefore, oncogenic signals are closely related to cell proliferation and transformation, which eventually leads to tumor production (Figure 4) [132].

### 7.4. Abnormal CGRP Target Gene Fragments Regulate Cell Stemness

Elevated promoter methylation level may be one of the most obvious characteristics for malignant tumors [157]. Many studies have showed that there are tumor-specific changes of DNA fragments in patients' serum with various malignant tumors, such as abnormal promoter methylation [158]. By real-time quantitative methylation-specific polymerase chain reaction (QMSP), it was found that the level of CALCA methylation in patients with lung cancer was significantly higher than that in benign lung lesions [157]. The high frequency of promoter methylation of CALCA gene has been found in cervical cancer and bladder cancer [159, 160]. Similarly, the previous study of our research group also found that the CpG island methylation of CALCA and CALCB in pancreatic ductal adenocarcinoma was significantly higher than that in adjacent tissues and CGRP participated in cell proliferation, apoptosis, differentiation, and survival through the AKT/CREB signaling pathway and finally promoted the occurrence and development of tumors [33]. CpG island hypermethylation of CALCA occurs in juvenile mononuclear leukemia (JMML), regaining the hypermethylated phenotype upon relapse after treatment [161]. Thus, the abnormal methylation level of CALCA fragment in the promoter region of stem cells is an important reason for the change of cell stemness [35], and high-methylation phenotype is also an important biomarker in the process of tumor diagnosis and treatment.

### 7.5. Transcription Factors Combined with GGAA Microsatellite Sequence Control CGRP Expression

Studies also confirmed that CALCB was a specific secretory peptide in Ewing's sarcoma (EWS). Silencing CALCB inhibited the growth of EWS cells, and the transcription factor ewsr1-fli1 combined with GGAA microsatellite sequence could regulate the expression of CALCB by improving enhancer activity, leading to the differentiation and proliferation of stem cells [132].

### 7.6. CGRP Gene Polymorphisms Increase Tumor Risk

CGRP has multiple gene polymorphic loci in tumors, and gene polymorphisms increase the risk of malignancy. It is known that extracellular calcium upregulates the proliferation of ovarian surface epithelial cells, which is very important for malignant transformation into ovarian cancer [162]. The CALCA gene codes for calcitonin, an important regulator of bone calcium metabolism. It was also reported that the T → C transition in base pairs upstream (-624) of the translation initiation codon of the CALCA gene increased risk of ovarian malignancy among Japanese (Figure 1(a)) [163].

Our previous study showed that promoter polymorphisms of CALCB gene (rs11603873 T/C and rs79501047 A/G) are common in the Han population, and the rs11603873 C genotype has a high risk of salivary adenoid cystic carcinoma compared with the rs11603873 T genotype, 2.27 times, and the rs79501047 G genotype was 3.76 times than that of the rs79501047 A genotype [164]. In a word, the polymorphism of *β*CGRP gene is related with genetic susceptibility to salivary adenoid cystic carcinoma, and serum CGRP and *β*CGRP can be used as new markers of salivary adenoid cystic carcinoma.

## 8. Summary

The 2021 Nobel Prize in physiology and medicine “temperature and tactile receptors” helps us perceive the world, feel temperature and pressure. CGRP has complex biological functions, and it is regulated by multiple cells and factors. Meanwhile, CGRP reacts on cells and other factors and helps maintain the stability of the body in the inflammatory state. Its complex shear mechanism provides the possibility for the different functional states in different tissues, and the adaptation is produced by organisms facing different environments during evolution. CGRP could regulate cell stemness during body damage repair and promote stem cell proliferation and differentiation, which has great potential application value in gene therapy. “New discoveries make life better,” manipulating CGRP may be able to controllably regulate stem cell differentiation, so as to realize the ultimate human dream of health and longevity.

## Data Availability

The data used to support the findings of this study are currently under embargo while the research findings are commercialized. Requests for data, 12 months after publication of this article, will be considered by the corresponding authors.
